# Sjögren Syndrome Associated with Inflammatory Muscle Diseases

**DOI:** 10.31138/mjr.29.2.92

**Published:** 2018-06-29

**Authors:** Michail P. Migkos, Ioannis Sarmas, George A. Somarakis, Paraskevi V. Voulgari, Konstantinos I. Tsamis, Alexandros A. Drosos

**Affiliations:** 1 Rheumatology Clinic, Department of Internal Medicine, Medical School, University of Ioannina, Ioannina Greece,; 2 Neurosurgical Institute, University of Ioannina School of Medicine, Ioannina, Greece; Department of Neurology, University Hospital of Ioannina, Ioannina, Greece

**Keywords:** Sjögren Syndrome, Inflammatory Myopathies, Inclusion Body Myositis, Polymyositis, Dermatomyositis

## Abstract

**Objectives:**

Sjögren’s syndrome (SS) is a chronic autoimmune inflammatory disorder characterized by diminished lacrimal and salivary gland function that may affect multiple organ systems. The association of SS with inflammatory myopathies (IM), a group of diseases characterized by chronic inflammation of striated muscle and skin has been infrequently described.

**Methods:**

We present two cases diagnosed with SS who developed IM. We have also conducted a review of the English literature to depict all available clinical evidence on the clinical association of SS with IM.

**Results:**

Two female patients diagnosed with SS developed polymyositis (PM) and inclusion body myositis (IBM) respectively. The literature review identified 24 cases with coexistence of the two autoimmune conditions (SS and IM). Twenty-two patients were females and two males. Eight patients were diagnosed with IBM, 15 were diagnosed with PM and 1 with dermatomyositis. All patients had biopsy proven IM.

**Conclusions:**

There is evidence of clinical association of primary SS and IM especially with IBM and PM. Patients with SS and symptoms of muscle weakness should be investigated for associated IM.

## INTRODUCTION

Sjögren Syndrome (SS) is a chronic autoimmune inflammatory disease that primarily affects the exocrine glands and has also a variety of extraglandular manifestations.^1–6^ SS can occur as a distinct disease entity (primary SS) or in the context of an underlying autoimmune rheumatic disease (secondary SS) mainly rheumatoid arthritis, systemic lupus erythematosus, scleroderma and others.^7–10^ Inflammatory myopathies (IM) is a heterogeneous group of disorders characterized by chronic inflammation of striated muscle and skin. They share features of skeletal muscle weakness and elevated serum levels of muscle enzymes.^11,12^ Muscle involvement is a frequent clinical manifestation in SS, expressed mainly with myalgias and muscle weakness. Frank IM is less common.^13–15^ In this report we present two cases of primary SS associated with IM with a literature review.

## METHODS

We present two cases of female patients diagnosed with Sjögren syndrome who were followed up regularly in the Rheumatology Department of the University Hospital of Ioannina. The patients developed muscle weakness and were additionally diagnosed with IM. We also reviewed the English language literature using PubMed database and the following index terms: SS, IM, inclusion body myositis (IBM), Polymyositis (PM), Dermatomyositis (DM) and myositis in order to indentify cases of SS associated with IM. We recorded the epidemiological features of the patients found in the literature including age, gender, age of diagnosis of SS and IM and their serological profile. All patients had to have positive muscle biopsy.

## CASE 1

A 70-year-old white female with a past medical history of arterial hypertension and sinus tachycardia was diagnosed with SS in 2002. The diagnosis was based on sicca symptoms (dry eyes and dry mouth), as well as positive objective sicca parameters for dry eyes (Schirmer’s I and Rose Bengal tests), positive antinuclear antibodies (ANA) at a titer of 1/320 with a fine speckled pattern, positive anti-Ro52 and minor salivary gland biopsy compatible with SS. The patient presented also arthralgias, thus she was treated with hydroxychloroquine (HCQ) 200mg/day. In August 2014 the patient complained of proximal and distal muscle weakness. The laboratory findings showed elevated serum muscle enzymes: creatine kinase (CK) 556 IU/L (normal range: 40–190 IU/L), lactate dehydrogenate (LDH) 371 U/L (normal range: 115–230 U/L). The transaminases (AST 32 IU/L, ALT 27 IU/L) and aldolase (3,8 IU/L) were in normal range. To rule out occult malignancy, a chest computed tomography and an ultrasound of the abdomen were performed and did not reveal significant findings. An electromyogram showed diffuse atrophy and loss of muscle fibers.

**Figure 1.**
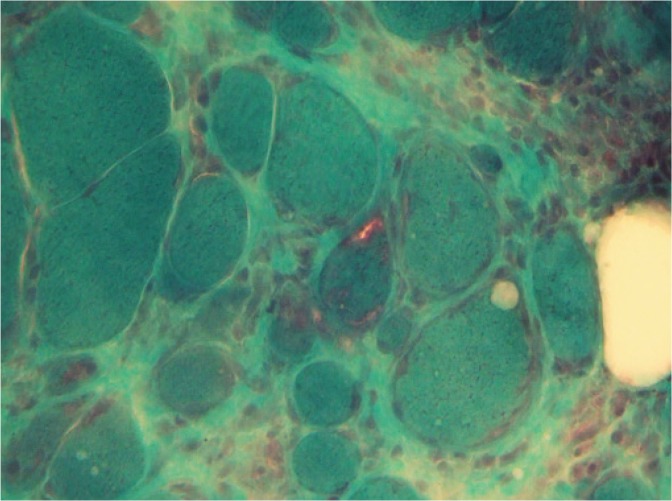
Muscle biopsy from a patient with SS and IBM (1^st^ Case). Modified Gomori trichrome. The muscle biopsy shows: pronounced variation in fiber size, inflammatory response and a fiber with rimmed vacuole

Muscle biopsy showed findings compatible with IBM (**Figure 1**). The patient started treatment with methylprednisolone 48mg/day with tapering and subsequently methotrexate (MTX) 15mg/week was added as a sparing agent of steroids. In May 2015 a new electromyogram revealed improvement of muscle findings which correlated with her clinical and laboratory improvement. To date the patient continues therapy with low doses of steroids (4mg/day) and MTX (15mg/week).

## CASE 2

**Figure 2.**
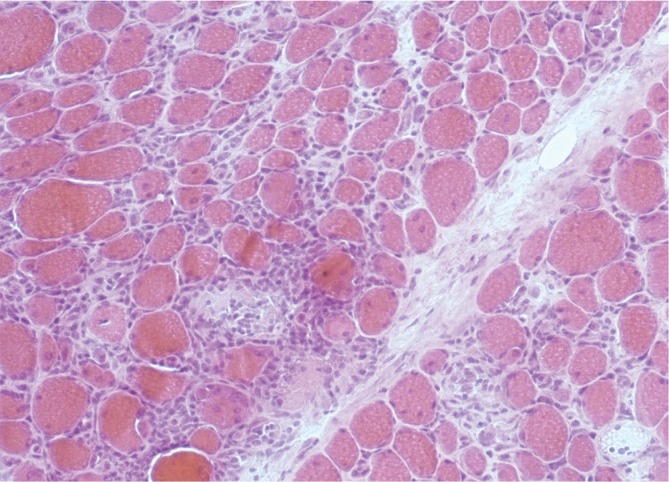
Muscle biopsy from a patient with SS and PM (2^nd^ Case). Haematoxylin & Eosin The muscle biopsy shows: endomysial inflammatory infiltrates

A 67-year-old white female was diagnosed in 2002 with SS based on arthralgias, sicca symptoms (dry eyes and dry mouth), positive ocular signs (Schirmer’s I and Rose Bengal tests), positive ANA at a titer of 1/320 with fine speckled pattern. In addition, minor salivary gland biopsy was positive for SS. In January 2015, she presented to our outpatient clinic with progressive proximal muscle weakness, inability to climb stairs and elevated muscle enzymes. Her past medical history included diabetes mellitus and dyslipidemia. She was treated with HCQ, atorvastastin, metformine, saxagliptin and pilocarpine. Her family doctor had discontinued HCQ and atorvastatin 10 months ago, when abnormal laboratory findings and clinical symptoms had begun. The patient was admitted to our clinic for further investigation. Physical examination revealed mild decrease of muscle strength of biceps and iliopsoas (4/5). Laboratory evaluation showed elevation of CK: 5004 IU/L, AST: 121 IU/L (normal range: 10–35 IU/L), ALT: 97 IU/L (normal range: 10–35 IU/L), LDH: 1059 IU/L and aldolase: 40 IU/L (normal range: 0–7,6 IU/L). An electromyogram showed significant features of IM while the muscle biopsy of the left quadriceps confirmed the diagnosis of PM (**Figure 2**). A computed tomography of the chest and abdomen was unremarkable. The patient was treated with methylprednisolone (48mg/day) and MTX (15mg/week) as a sparing agent of steroids. Six months later, the patient had significant clinical (muscle strength 5/5) and laboratory (AST: 16 IU/L, ALT: 97 IU/L, CK: 103 IU/L, LDH: 451 IU/L) improvement, while the dose of steroids was decreased to 4mg/day and the dose of MTX was stable.

## DISCUSSION

According to the literature the percentage of patients with primary SS who manifested symptoms of IM, widely vary between 2,5% – 10%.^16^ Most studies showed significantly lower percentages of patients with primary SS who developed true IM (proven by biopsy). In the study of Colafrancesco et al. the percentage of the SS patients with manifestations of biopsy proven myositis was 0,75% (10 out of 1320).^17^ Another recent study published by Kanellopoulos et al. demonstrated 3 out of 518 (0,6%) patients with primary SS who were diagnosed with myositis.^18^ The study of Kraus et al raised this rate to 3%.^13^ Both our cases had SS fulfilling the American European consensus group criteria^19^ and both developed IM many years after the diagnosis of SS. In our first case the patient was diagnosed with IBM 13 years after the diagnosis of primary SS. Eight cases of SS patients, diagnosed according the performed biopsy, with IBM were reported (**Table 1**). The interval between the two diagnoses largely varied from 0 to 14 years. In the majority of the patients (7/8) ANA were positive. Anti Ro/SSA antibodies were positive in half of them (4/8). Anti La/SSB antibodies were positive only in one patient, while RF was positive in 5 out of 8 patients. In our case the patient was positive for ANA and anti-Ro/SSA antibodies.

**Table 1:** Published cases of primary Sjogren Syndrome associated with inflammatory myopathies (proven by biopsy).

**Author (year)**	**Study**	**Sex**	**Age at onset of pSS**	**Age at onset of IM**	**Classification of IM (Biopsy)**	**Autoantibodies**
Chad D. et al. (1982)	Case report	F	32	33	IBM	RF, ANA
Ringel SP. et al. (1982)	Case series (1/4)	F	64	64	DM	RF, ANA, Ro/SSA, La/SSB
Ringel SP. et al. (1982)	Case series (2/4)	F	42	42	PM	RF, ANA, Ro/SSA, La/SSB
Ringel SP. et al. (1982)	Case series (3/4)	F	55	55	PM	RF, ANA, Ro/SSA, La/SSB
Ringel SP. et al. (1982)	Case series (4/4)	F	29	29	PM	ANA, Ro/SSA, La/SSB
Gutmann L. et al (1985)	Case report	F	58	60	IBM	RF, ANA
Leroy JP. et al. (1990)	Case report	F	65	65	PM	RF, ANA, Ro/SSA, La/SSB
Khraishi MM. et al (1992)	Case report	F	-	69	IBM	RF, ANA, Ro/SSA, La/SSB
Imasaki T. et al. (1996)	Case report	F	53	53	PM	-
Kanellopoulos P. et al. (2002)	Case series (1/3)	F	33	47	IBM	ANA, AMA, Ro/SSA
Kanellopoulos P. et al. (2002)	Case series (2/3)	M	67	66	IBM	ANA, Ro/SSA
Kanellopoulos P. et al. (2002)	Case series (3/3)	F	52	45	IBM	ANA, Ro/SSA
Aoki A. et al. (2003)	Cohort study (1/5)	F	74	74	PM	ANA, Ro/SSA, La/SSB
Aoki A. et al. (2003)	Cohort study (2/5)	F	63	63	PM	ANA, Ro/SSA, RNP
Aoki A. et al. (2003)	Cohort study (3/5)	F	62	-	PM	RF, ANA, Ro/SSA, RNP
Aoki A. et al. (2003)	Cohort study (4/5)	F	57	57	PM	ANA, Ro/SSA, La/SSB
Aoki A. et al. (2003)	Cohort study (5/5)	F	50	50	PM	ANA
Misterska-Skora M et al. (2013)	Case report	F	47	47	IBM	RF, ANA
Colafrancesco S. et al. (2015)	Cohort study (1/6)	F	46	61	PM	RF, ANA, Ro/SSA, La/SSB
Colafrancesco S. et al. (2015)	Cohort study (2/6)	F	57	58	PM	-
Colafrancesco S. et al. (2015)	Cohort study (3/6)	F	68	69	IBM	RF
Colafrancesco S. et al. (2015)	Cohort study (4/6)	F	62	52	PM	RF, ANA, Ro/SSA,
Colafrancesco S. et al. (2015)	Cohort study (5/6)	F	37	42	PM	RF, ANA, Ro/SSA, La/SSB, Jo1, RNP
Colafrancesco S. et al. (2015)	Cohort study (6/6)	M	53	53	PM	ANA, Ro/SSA, Jo1
Migkos MP. et al. (Present study)	Case series (1/2)	F	57	70	IBM	ANA, Ro/SSA
Migkos MP. et al. (Present study)	Case series (2/2)	F	54	67	PM	ANA

pSS: primary Sjogren syndrome, IM: inflammatory myopathies, DM: dermatomyositis, PM: polymyositis, IBM: inclusion body myositis, ANA: antinuclear antibodies, RF: rheumatoid factor, M: male, F: female

IBM differentiates its clinical presentation from the other IM. IBM affects predominantly the distal muscles and more specific the foot extensors and finger flexors. IBM is usually refractory to treatment. Corticosteroids and immunosuppressive drugs (MTX, azathioprine, cyclosporine) have failed to show long term benefits.^12,20^ Despite the transient effect of intravenous immune globulin, it remained ineffective in controlled trials.^21,22^ IBM associated with SS responded better to treatment compared to patients diagnosed with IBM alone. In the study by Colafrancesco et al, one patient diagnosed with IBM was treated with intravenous immune globulin and achieved clinical and laboratory remission.^17^ In a case report with biopsy proven IBM reported by Misterska-Skora et al., the patient was treated with steroids and MTX and achieved remission in 8 months.^23^ Kanellopoulos et al published three cases with primary SS and IBM. Two of them were treated with HCQ and 1 received steroids and MTX. All three patients showed improvement in their muscle strength.^18^ In our case the patient also received corticosteroids and MTX and had significant clinical and laboratory improvement 8 months after treatment.

PM is also associated with primary SS. In the literature review we identified 15 cases of primary SS and biopsy proven PM (**Table 1**). Thirteen out of 15 patients had positive ANA. Anti-Ro/SSA antibodies were present in 12 patients, while anti-La/SSB antibodies and rheumatoid factor was present in 8 and 7 out of 15 patients respectively. In our second case the patient had only positive ANA. Aoki et al presented 5 cases of PM associated with primary SS. The patients received prednisolone and they achieved clinical and laboratory remission. In three of them, relapse of the disease was mentioned with muscle weakness and elevation of CK. In those patients the dose of corticosteroids was increased, and remission was achieved without additional immunosuppressive therapy.^24^ Colafrancesco et al diagnosed 5 patients with PM. All patients were treated with steroids and HCQ and according to their response pulses cyclophosphamide and rituximab were used for achieving remission.^17^ Pulses of cyclophosphamide therapy were also used by Leroy et al in a patient with SS and PM.^15^ Finally, Imasaki et al described a patient with bronchiolitis obliterans organizing pneumonia, primary SS and PM. The patient received prednisolone and two months later achieved remission.^25^ In our second case, the patient was diagnosed with PM and received methylprednisolone and MTX successfully. Only one case with biopsy-proven DM in a patient with SS was identified in the literature.^26^

Additionally, it should be mentioned that in rare cases of myositis associated with SS, with CK and electromyography abnormalities, muscle biopsy and histology examination were mandatory, in order not to overlook a real PM-overlap syndrome, misinterpreted as extraglandular involvement of SS.^27^

On the other hand, it is mandatory in patients with SS who have developed IM, to rule out other disorders known to cause myopathy. To this end, one must exclude lymphoma development, statin and HCQ induced myopathy. In the first case, a careful clinical examination with the appropriate imaging tests, tissue biopsy, as well as the detection for cryoglobulins, measurement of complement levels and serum electrophoresis will help the diagnosis of lymphoma.^28^ In the cases of statin and HCQ-induced myopathy, a detail past medical history, the presence of antibodies against 3 –hydroxy-3 methylglutaril-coenzyme-A reductase (HMGCR), the enzyme target of statin therapy and muscle biopsy will differentiate these two entities from IM. It is reported that statin myopathy is associated with the presence of HMGCR antibodies and causes a necrotizing myopathy in the affected muscles.^29,30^ In addition, HCQ-induced myopathy is a rare condition which may cause proximal muscle weakness and cardiomyopathy and the muscle biopsy shows curvilinear bodies and vacuolar changes.^31^

Finally, novel studies highlight that anti-Ku antibodies have been reported in a wide spectrum of autoimmune diseases, sometimes in association with IM. In the study of Rigolet et al, out of 34 anti-Ku positive patients, 11 had IM, 8 of them as part of an overlap syndrome defined as IM associated with connective autoimmune disease (systemic sclerosis, SS and systemic lupus erythematosus).^32^ In a recent study published by Wielosz et al are reported 3 cases of anti-Ku positive patients: it concludes that the presence of anti-Ku antibodies is associated with a wide range of non-specific symptoms regarding muscle, joint and skin involvement (33). In another recent study published by Fiorentino et al, a new 60 kDa specificity was detected by immunoblotting HeLa cell lysates, the targeted autoantigen was identified as poly(U)-binding-splicing factor 60 kDa (PUF60). In this study it is reported that the new antibody anti-PUF60 it is present in SS patients with DM.^34^

## CONCLUSION

Patients with primary SS and symptoms of muscle weakness should be investigated for associated IM. There is evidence of clinical association of primary SS and IM especially with IBM and PM. It seems that both entities respond better to treatment when they are associated with SS than as district entities.
